# A structured, telephone-delivered intervention to reduce methamphetamine use: study protocol for a parallel-group randomised controlled trial

**DOI:** 10.1186/s13063-023-07172-9

**Published:** 2023-03-29

**Authors:** Dan I. Lubman, Victoria Manning, Shalini Arunogiri, Kate Hall, John Reynolds, Peta Stragalinos, Rachel Petukhova, Robyn Gerhard, Jonathan Tyler, Anna Bough, Anthony Harris, Jasmin Grigg

**Affiliations:** 1 Turning Point, Eastern Health, Melbourne, Victoria Australia; 2 Monash Addiction Research Centre, Eastern Health Clinical School, Monash University, Melbourne, Victoria Australia; 3 School of Psychology, Deakin University, Geelong, Victoria Australia; 4 Centre for Drug use, Addictive and Anti-social Behaviour Research, Deakin University, Melbourne, Victoria Australia; 5 Faculty of Medicine, Nursing and Health Sciences, Monash University, Melbourne, Victoria Australia; 6 Centre for Health Economics, Monash University, Melbourne, Victoria Australia

**Keywords:** Methamphetamine, Substance use disorder, Treatment, Psychological intervention, Telehealth, Randomised controlled trial

## Abstract

**Background:**

Australia has one of the highest rates of methamphetamine (MA) use in the world; however, uptake of in-person psychological treatment remains extremely low due to numerous individual (e.g. stigma, shame) and structural (e.g. service accessibility, geographical location) barriers to accessing care. Telephone-delivered interventions are ideally placed to overcome many of the known barriers to treatment access and delivery. This randomised controlled trial (RCT) will examine the efficacy of a standalone, structured telephone-delivered intervention to reduce MA problem severity and related harms.

**Methods:**

This study is a double-blind, parallel-group RCT. We will recruit 196 ± 8 individuals with mild to moderate MA use disorder from across Australia. After eligibility and baseline assessments, participants will be randomly allocated to receive either the Ready2Change-Methamphetamine (R2C-M) intervention (*n* = 98 ± 4; four to six telephone-delivered intervention sessions, R2C-M workbooks and MA information booklet) or control (*n* = 98 ± 4; four to six ≤5-min telephone check-ins and MA information booklet including information on accessing further support). Telephone follow-up assessments will occur at 6 weeks and 3, 6 and 12 months post-randomisation. The primary outcome is change in MA problem severity (Drug Use Disorders Identification Test, DUDIT) at 3 months post-randomisation. Secondary outcomes are as follows: MA problem severity (DUDIT) at 6 and 12 months post-randomisation, amount of methamphetamine used, methamphetamine use days, methamphetamine use disorder criteria met, cravings, psychological functioning, psychotic-like experiences, quality of life and other drug use days (at some or all timepoints of 6 weeks and 3, 6 and 12 months post-randomisation). Mixed-methods program evaluation will be performed and cost-effectiveness will be examined.

**Discussion:**

This study will be the first RCT internationally to assess the efficacy of a telephone-delivered intervention for MA use disorder and related harms. The proposed intervention is expected to provide an effective, low-cost, scalable treatment for individuals otherwise unlikely to seek care, preventing future harms and reducing health service and community costs.

**Trial registration:**

ClinicalTrials.gov NCT04713124. Pre-registered on 19 January 2021.

**Supplementary Information:**

The online version contains supplementary material available at 10.1186/s13063-023-07172-9.

## Background and rationale

Methamphetamine (MA) use is a key contributor to the burden of disease in Australia and globally [1]. Approximately 1.2 million (5.8%) Australians have used MA in their lifetime, with 1.3% reporting recent use (i.e. last 12 months), making this potent central nervous system (CNS) stimulant the most commonly used illicit drug after cannabis and cocaine [2]. Wastewater population estimates show Australia’s annual consumption of MA was increasing year on year until 2020 when COVID-19 lockdowns and border closures caused disruption to MA supply in Australia [3].﻿ Despite this, MA remains the drug with the most problematic use in Australia with 77% of the estimated expenditure in major illicit drug markets spent on MA [4]. Along with upward trends in purity and potency, regular (i.e. daily or weekly) use has more than doubled in under a decade to 20% of MA consumers in 2016 [2], while the proportion of injecting use has doubled in 3 years, to 19% of current consumers in 2016 [2], and the rate of MA dependence now among the highest globally [5]. These trends in MA use have been accompanied by a visible pattern of increasing severe physical and psychological harms and significant public health and social consequences [6, 7].

However, the rate at which individuals with MA use disorder seek help in mainstream treatment services remains extremely low [8], due to a range of individual barriers that include experiences of shame and stigma, attitudes toward in-person treatment and readiness for change and structural barriers that include service accessibility (e.g. wait lists, service operating hours), geographical location and time constraints [6]. Additionally, national data has identified a significantly higher prevalence of MA use in regional and remote areas (2.5 times greater than in major cities) [2, 3, 9], where there are proportionately far fewer episodes of treatment received by people living in these locations [9], and the multiple barriers to accessing treatment are heightened: anonymity is harder to achieve in smaller communities; individuals can face increased stigmatisation and discrimination; there are fewer treatment services; and service location and poor public transport options can make treatment access prohibitive [10].

Current evidence-based treatment options for MA are limited. To date, there are no approved pharmacotherapies for the treatment of MA use disorder, with pharmaceutical treatments so far failing to exhibit substantive and consistent effects [11]. Cognitive and behavioural interventions currently represent the gold standard treatment for MA use disorder and are systematically shown to reduce MA use, increase abstinence and improve treatment adherence [12, 13]. Even among regular MA consumers, as little as two intervention sessions have been found to have positive effects on MA use [14]. Additionally, there is increasing evidence that models of addiction treatment combining two or more psychosocial approaches (e.g. motivational interviewing, cognitive behavioural therapy) provide even stronger, additive treatment effects, including improved psychological health [15, 16]. While multicomponent, multi-session, integrated psychological interventions currently represent the best treatment option for MA use disorder [17, 18], the large-scale impact of these treatments remains limited as they require substantial investments in healthcare delivery systems and adequate cover across jurisdictions.

A key approach to facilitate earlier treatment among individuals with MA use disorder who do not seek help in traditional settings is to offer treatment in alternative, more accessible formats. Telephone-delivered interventions are convenient, flexible and permit a sense of privacy and anonymity [19], providing a mechanism for treatment delivery that overcomes many of the known barriers to accessing treatment, and can more easily capitalise on fleeting motivation to enter treatment [20, 21]. Telephone-delivered interventions can also be used within a stepped care model, where individuals commence work on reducing their substance use before engaging in longer, more intensive programs. While individuals with severe MA use disorder usually require more intensive treatment, telephone-delivered models provide a novel opportunity for engagement in the continuum of treatment and may serve to prevent the development of more severe MA-related problems for individuals with mild to moderate MA use disorders.

Current research on the benefits of telephone-delivered interventions for smoking cessation [22], alcohol use disorder [19, 20, 23, 24], and some illicit drug use disorders (e.g. cocaine, cannabis) supports continued exploration into telephone-delivered alcohol and other drug (AOD) interventions [21]. Equivalence of telephone-delivered and in-person AOD interventions has been demonstrated using metrics of therapeutic alliance [19, 25], abstinence supported by urinalysis data [25, 26] and treatment retention rates [27]. This study will be the first randomised controlled trial (RCT) conducted internationally to examine the effects of a standalone telephone-delivered intervention in reducing MA problem severity and related harms.

### Objectives

The aim of this study will be to examine the efficacy of a structured, telephone-delivered intervention, Ready2Change-Methamphetamine (R2C-M), in reducing MA problem severity among individuals with mild to moderate MA use disorder (as defined by Structured Clinical Interview for DSM-5 Research Version (SCID-5-RV) [28]), compared with a minimal input control condition of weekly telephone check-ins, and a MA information booklet including information on accessing further support.

### Trial outcomes

**Fig. 1:**
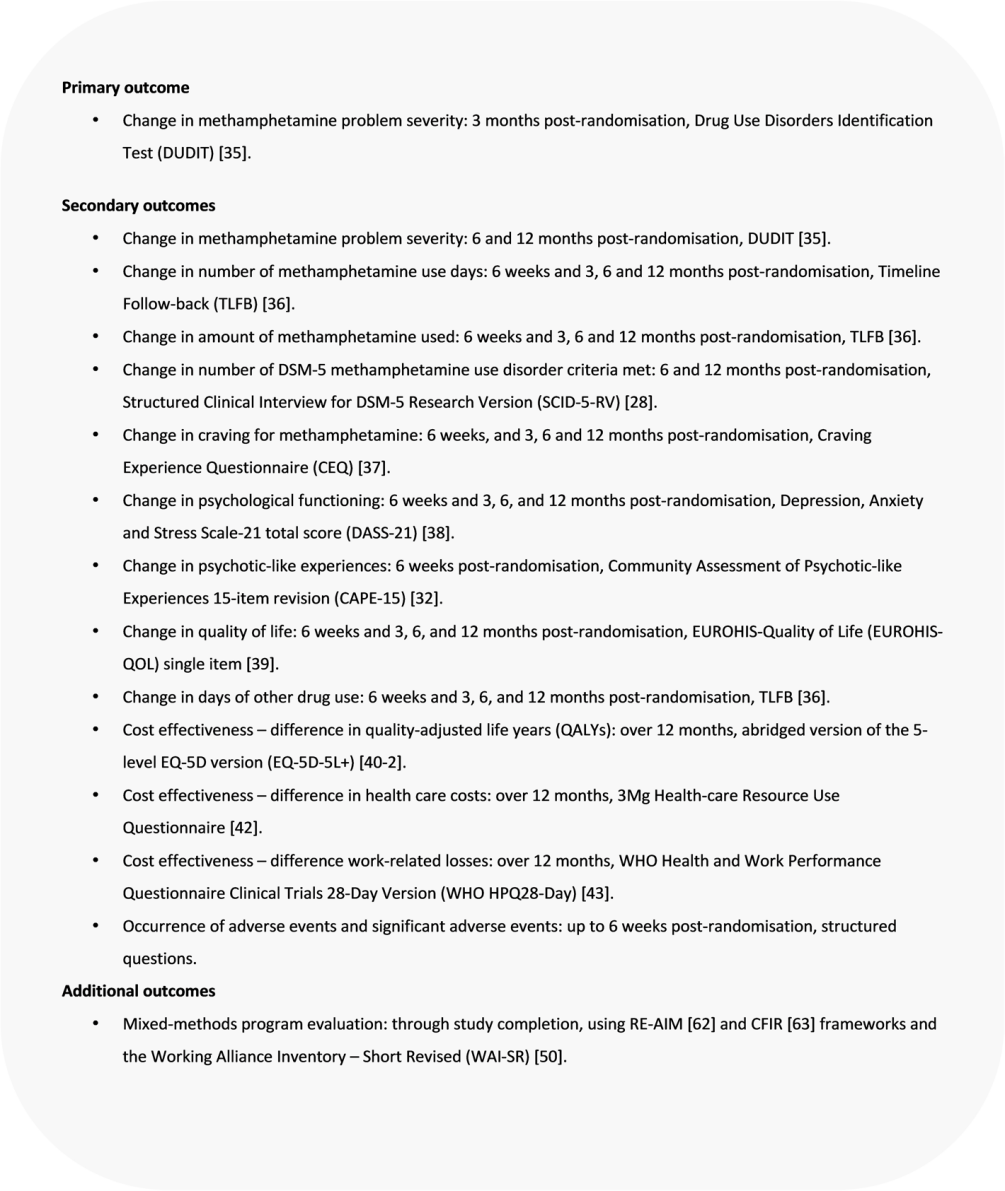
Primary and secondary outcomes

Primary and secondary outcomes for this trial are detailed in Fig. 1.

## Methods

### Trial design

**Fig. 2:**
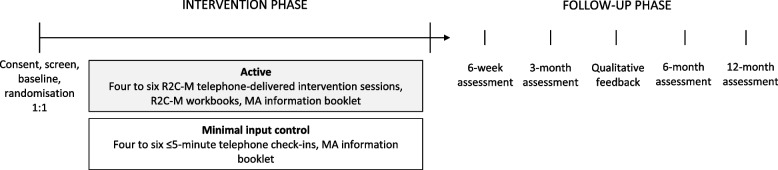
Study design

This study is a single-site, double-blind, parallel-group, superiority RCT, with participants randomly allocated to receive either R2C-M intervention or a minimal input control at a 1:1 allocation ratio (Fig. 2). The protocol follows Standard Protocol Items: Recommendations for Interventional Trials (SPIRIT) guidelines (Table 3; see Additional file 1: SPIRIT Checklist) [29].

### Study setting

The trial will be based within Turning Point’s Telephone and Online Treatment Services, Melbourne, Australia. All intervention and assessment elements of participation will be conducted via telephone. Turning Point operates multiple helplines across several jurisdictions (>100,000 contacts per annum), as well as two national online counselling services (>1 million page views per annum). Turning Point (in collaboration with Monash and Deakin Universities) supported the development of the R2C program and has tested its benefits among individuals with alcohol, methamphetamine and cannabis use problems by way of two non-controlled studies [20, 24] and a controlled trial of effectiveness among people with alcohol use disorder [30, 31].

### Participants

A total of 196 ± 8 participants, allowing for variation in post-3 month assessment attrition rates and for the randomisation of participants who have commenced pre-eligibility at the time accrual is about to close, will be randomly allocated to one of the two intervention conditions (i.e. 98 ± 4 participants per trial arm).

### Eligibility

Inclusion and exclusion criteria are shown in Table 1.

**Table 1:** Inclusion and exclusion criteria

**Inclusion criteria**	
• Age 18+ years • Mild or moderate MA use disorder (DSM-5 diagnosis confirmed at eligibility assessment using the *Structured Clinical Interview for DSM-5 Research Version*, SCID-5-RV) [28] • Used MA on at least two occasions in the past month • Seeking to reduce MA use • Able to provide informed consent and comply with the requirements of the treatment protocol • Willing to provide the contact details of their general practitioner or other treating physician, if available, for follow-up • English as a first language or fluent • Educated to high school level (literacy) • Regular access to a telephone • Postal/email address to receive intervention materials	
**Exclusion criteria**	
• Currently receiving treatment for substance use disorder (e.g. medically supervised detoxification, residential rehabilitation, drug counselling, pharmacotherapy—this criterion applies only at trial enrolment and does not preclude the participant from entering treatment/receiving usual care during the trial) • Requiring acute care for severe substance use disorder (DSM-5 diagnosis confirmed at eligibility assessment using the SCID-5-RV [28] with oversight from the principal investigator or study clinician) • Requiring acute care for active suicidality or unstable psychiatric condition • A diagnosed primary psychotic disorder (schizophrenia, schizoaffective disorder, bipolar disorder) • Pregnancy • Hearing impairment that would prohibit participation in telephone intervention / follow-up assessments	

### Recruitment and eligibility

This study will use multiple channels to recruit a representative sample of individuals with mild-to-moderate MA use disorder from across Australia, including (i) online and social media advertising, (ii) alcohol and other drug (AOD) helplines that do not offer the R2C program, (iii) GP referrals, and (iv) via opportunistic study promotion (e.g. hospital/community newsletters, dissemination of study brochures/posters). Individuals who respond to study advertising will be directed to a secure Qualtrics® form where some brief pre-eligibility questions are asked about (i) prior barriers to seeking AOD treatment and (ii) current substance use problem severity. Individuals identified from the pre-eligibility questions as having substance use problem severity that is very low or very high will not progress to receive a call-back, but will instead be thanked for their time and provided with the contact details of their state-specific AOD counselling, referral and information helpline as well as the national Counselling Online service. Individuals identified from the pre-eligibility questions as having MA problem severity that may meet inclusion criteria will receive a call-back from the research team (Researcher 1).

During initial contact, Researcher 1 will provide potential participants with an overview of the study (aims, procedures, risks and benefits), provide the Participant Information Sheet (email/mail; Additional file 2) and respond to any questions. Participants will be asked to provide their informed verbal consent for their re-identifiable data to be used in future, related research projects during the eligibility assessment. No other consent provisions (e.g. for biological specimens) are necessary. The eligibility assessment can be undertaken during the initial phone call or scheduled for another time, as preferred by the participant. Eligibility will be assessed using the eligibility measures presented in Table 2, with data entered into the trial’s REDCap (Research Electronic Data Capture) [51] data collection form by Researcher 1. A baseline call will be scheduled with individuals who are deemed eligible to participate; individuals who do not meet the study participation criteria will be offered a referral to a local AOD service, identified via the appropriate Alcohol and Drug Information Service (ADIS) and/or a service navigator tool.

**Table 2:** Trial measures

Data collected	Method
***Eligibility measures***	
Pre-eligibility questions	Brief information on prior barriers to seeking AOD treatment and current substance use problem severity collected using structured questions.
Demographic information	Demographic information (e.g. age, gender, education level) collected using structured questions.
SCID-5-RV	Presence and severity of MA use disorder assessed using the Structured Clinical Interview for DSM-5 Research Version (SCID-5-RV) [28] in combination with clinical review. Scores range 0–11, with higher scores suggesting greater severity of MA use disorder. Scores ≤1 or ≥ 6 typically warrant clinical review for inclusion in the study. The SCID-5-RV will also be used as a measure of change in this trial.
CAPE-15	Psychotic-like experiences assessed using the Community Assessment of Psychotic-like Experiences, 15-item revision (CAPE-15) [32]. Score range 1–4 for frequency and 1–4 for distress, with higher scores indicating greater symptom frequency and distress. Scores >1.47 (cut-off value for people at ultra-high risk of psychosis [33]) will be reviewed in conjunction with information on recent psychiatric- or AOD-related hospitalisations and current medications. The CAPE-15 will also be used as a measure of change in this trial.
SIDAS	Suicidal risk assessed with the Suicidal Ideation Attributes Scale (SIDAS) [34]. Scores >21 indicate a high risk of suicidal behaviour. Further structured clinical questions will be asked when required to assess risk.
Other eligibility	Structured questions assess additional inclusion/exclusion criteria (e.g. current AOD treatment, diagnosed primary psychotic disorder).
***Primary outcome***	
DUDIT	MA problem severity assessed with the Drug Use Disorders Identification Test (DUDIT) [35] at 3 months post-randomisation. Scores range 0–44. Higher score suggests more severe MA use problem. The DUDIT will also be used as a secondary outcome measure (6 and 12 months post-randomisation). The time frame has been adapted to cover month prior to assessment (rather than year), so that planned follow-up assessments can be performed.
***Secondary outcomes***	
TLFB	Days of MA use, amount of MA used and days of other drug use in past 28 days assessed with the Timeline Follow-back (TLFB) calendar-based assessment tool [36].
CEQ	Past-week frequency of MA cravings, and strength of strongest craving, assessed with the Craving Experience Questionnaire (CEQ) [37]. Scores range from 0 to 100. Higher score indicates greater craving frequency and strength.
DASS-21	Past-month psychological functioning assessed with the Depression, Anxiety and Stress Scale-21 (DASS-21) [38]. Total scores range from 0 to 63 (depression scored 0–21, anxiety scored 0–21, stress scored 0–21). Higher score indicates higher symptom severity.
EUROHIS-QOL single item	Past-month quality of life (QoL) assessed with the EUROHIS-QOL single item [39].
AEs	Adverse events monitored with structured questions relevant to MA use disorder cohort and trials of psychotherapeutic interventions.
***Cost-effectiveness***	
EQ-5D-5L+	Quality-adjusted life years (QALYs) assessed with the EuroQol, 5 dimensions, 5 levels (EQ-5D-5L+) [40, 41].
3Mg trial’s Health-care Resource Use Questionnaire	Health resource usage in past 3 months assessed with the 3Mg trial’s Health-care Resource Use Questionnaire [42].
WHO HPQ28-Day	Time lost from work or from lower work productivity assessed with the WHO Health and Work Performance Questionnaire Clinical Trials 28-Day Version (WHO HPQ28-Day) [43].
***Additional measures***	
SBQ	Barriers to help-seeking for MA use disorder assessed with the Short Barriers Questionnaire (SBQ) [44]. Scores range from 0 to 66 (low perceived need scored 0–27; stigma scored 0–18; apprehension scored 0–21). Higher scores indicate greater importance of barrier.
RR-ICR	Readiness to change at randomisation assessed with the Readiness Ruler I-C-R (RR-ICR) [45]. Importance, confidence and readiness scored 0–10. Higher scores indicate greater change readiness. The RR-ICR will be used as a predictor of treatment response in this trial.
CIS	Impulse control assessed with the Cognitive Impulsivity Suite (CIS) [46] in a subsample of participants willing to complete the additional task.
Sleep measures	Chronotype assessed with the Reduced Morningness-Eveningness Questionaire (rMEQ) [47, 48], and sleep quality and disturbances assessed with the Pittsburgh Sleep Quality Index (PSQI) [49].
Mixed-methods program evaluation	Program reach assessed by response rate to trial advertising, participant engagement (i.e. number of sessions completed), reach to rural and regional areas and health inequity groups (i.e. participation rates by gender, Aboriginal and Torres Strait Islander status, lesbian, gay, bisexual, transgender, intersex, queer, and other LGBTIQ+ status, and culturally and linguistically diverse background, disability status). R2C-M program feedback via participant qualitative interviews and the Working Alliance Inventory – Short Revised (WAI-SR) [50] with ~30% of participants allocated to the intervention condition.

*MA* methamphetamine

### Baseline and randomisation

Participant-reported outcome measures will be administered at baseline (Tables 2 and 3), immediately after which participants will be randomly assigned to R2C-M or control group with a 1:1 allocation ratio (day 1). Randomisation will be stratified by gender—(i) female, (ii) male and (iii) self-described/prefer not to say—and will use a standard computer-generated “permuted blocks of variable size” scheme for each stratum. Randomisation lists for each stratum will be generated at the start of the study by the trial statistician and linked to a unique identification code. The statistician who prepares the lists will play no other role in the delivery of the interventions. Allocations will be contained in the trial’s REDCap electronic data form/participant registration system. At the completion of baseline data collection, Researcher 1 will perform randomisation using the REDCap randomisation function and will inform the participant of the trial arm they have been allocated to. Researcher 1 will not be involved in follow-up data collection and therefore does not need to be blind to participant allocation. Participants, the researcher collecting follow-up data for the trial (Researcher 2) and the statistician will remain blind to participant allocation. A procedure for unblinding is not necessary as, in the case of a serious adverse event (SAE) where unblinding may be necessary, individuals in contact with the participant and/or who will respond to a SAE (e.g. principal investigator, study clinician, R2C-M counsellor) will not be blinded [34, 44, 46].

### Intervention

**Fig. 3:**
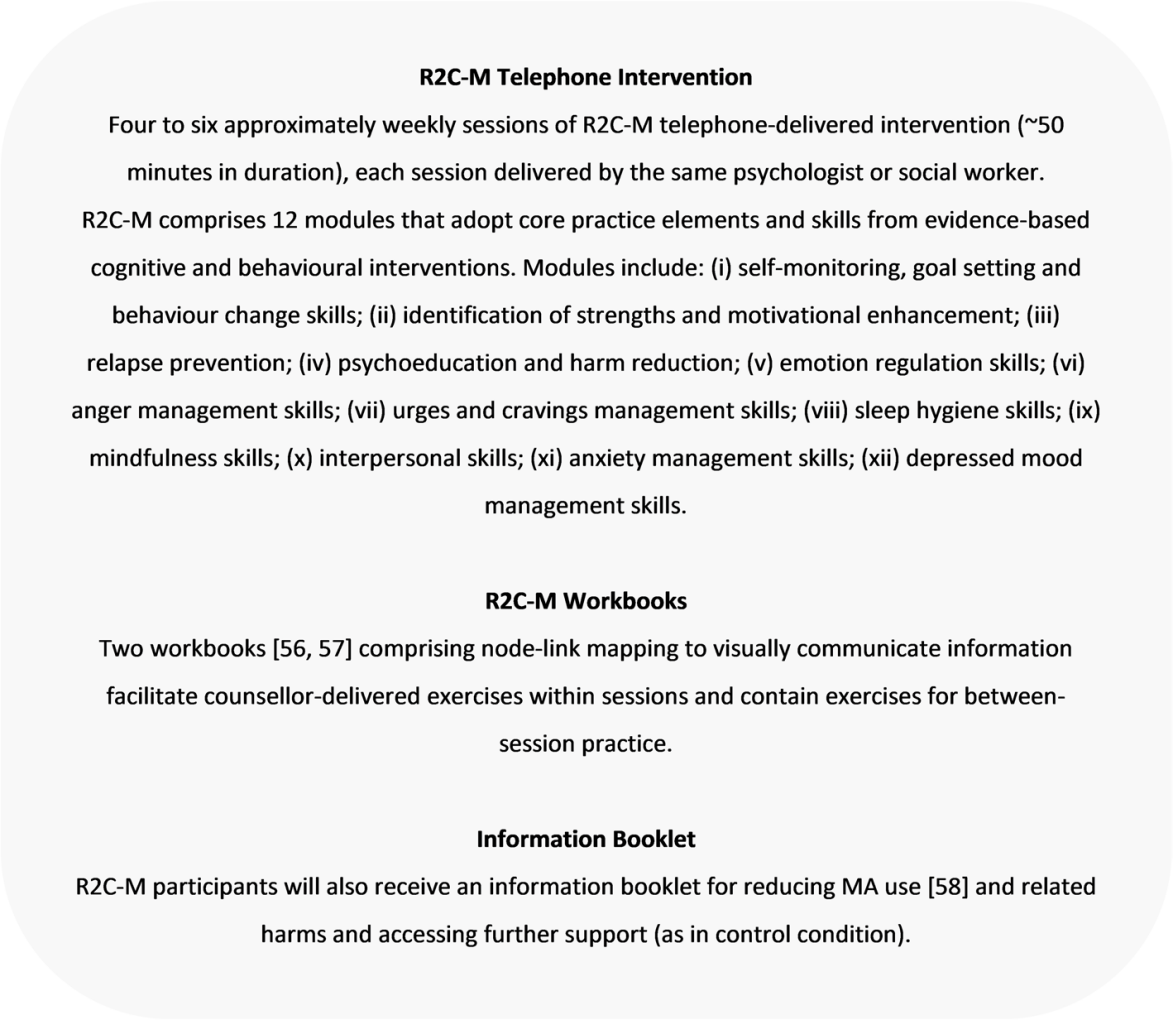
R2C-M intervention condition

Participants randomised to the R2C-M intervention will be contacted approximately weekly to receive four to six sessions of the R2C-M telephone-delivered intervention (typically 50 min in duration, delivered by the same counsellor), a manualised intervention comprising 12 modules that adopt core practice elements from evidence-based interventions including motivational interviewing [52], cognitive behavioural therapy [53], relapse prevention [54] and acceptance and commitment therapy [55], which are delivered flexibly according to clients’ individual needs (Fig. 3). Two R2C-M workbooks comprising node-link mapping to visually communicate information are mailed/emailed to clients to facilitate counsellor-delivered exercises within sessions and contain self-help exercises for between-session practice [56, 57]. An information booklet for reducing MA use and related harms, and accessing further support, will also be provided (as in control condition) [58] (Fig. 3). R2C-M counsellors on the study will be psychologists or qualified social workers trained by CI Hall, who led the development of the R2C intervention.

**Fig. 4:**
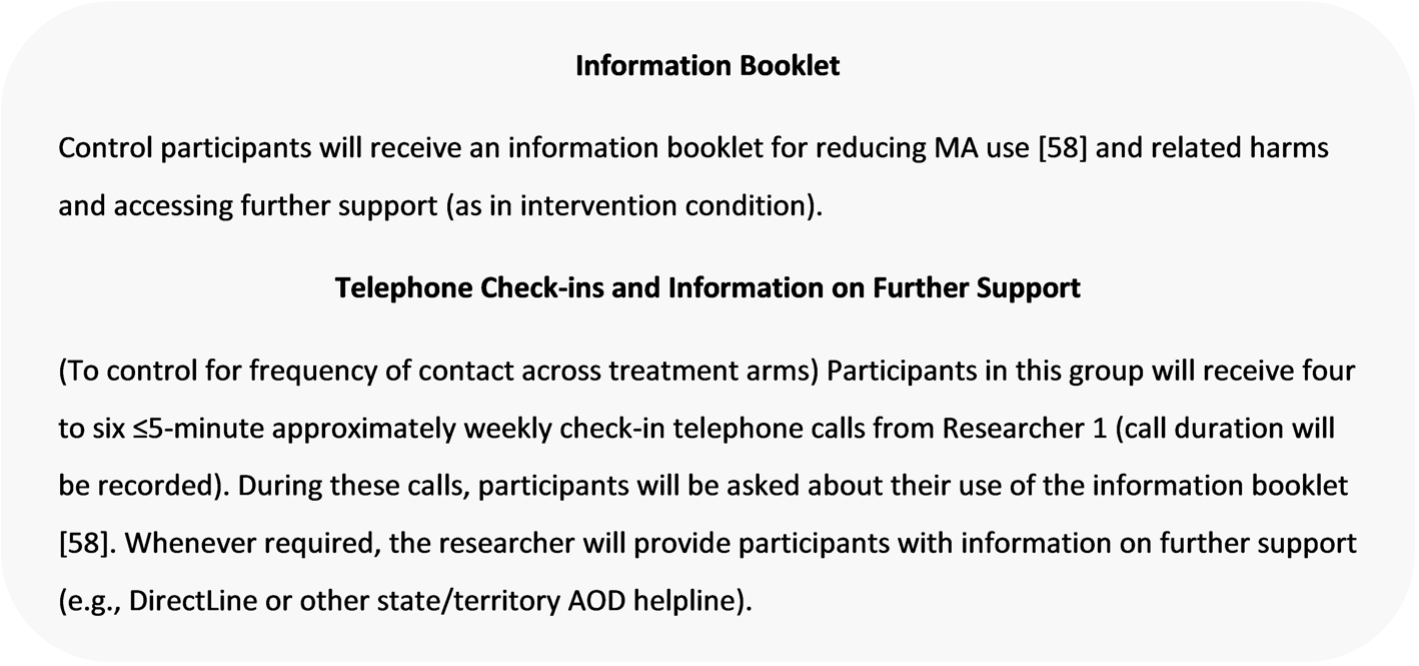
Control condition

Participants randomised to the control condition will receive the MA information booklet (as in the intervention condition) and four to six ≤5-min approximately weekly check-in telephone calls (from Researcher 1), in which participants will be asked about their use of the booklet and can be provided information on further supports (e.g. state/territory AOD helpline) (Fig. 4). Call duration will be recorded for both R2C-M and control check-in calls [56–58].

#### Choice of comparator

Research suggests that even baseline questioning and brief health education/advice can yield positive short-term changes in AOD use, by prompting reflection, self-regulation of behaviour and treatment-seeking [59–61]. In addiction treatment research, control groups have comprised no treatment, treatment as usual (e.g. case management) or “minimal input” comparators. In this trial, the information booklet for reducing MA use and related harms, and accessing further support [58], as well as four to six ≤5-min approximately weekly telephone check-ins to control for frequency of contact across treatment arms, is considered to be a minimal input control condition. While this condition may impact positively on participants’ MA use, we expect a more modest benefit relative to the R2C-M intervention condition.

#### Timeline

See the timeline in Table 3.

**Table 3:** SPIRIT table schedule of enrolment, interventions and assessments

	Study period							
	Pre-trial	Eligibility	Baseline	Intervention	Follow-up			
Timepoint		-1wk	Day 1	1-6wk	6wk	3mth	6mth	12mth
***Enrolment***								
Pre-eligibility questions	x							
Informed consent		x						
Demographic and eligibility questions		x						
SCID-5-RV		x					x	x
CAPE-15		x			x			
SIDAS + additional questions		x						
AOD treatment enquiry		x			x	x	x	x
***Intervention assignment***								
Randomisation			x^*b*^					
***Intervention***								
R2C-M sessions								
Control check-ins								
***Outcome measures***								
DUDIT		x				x^*a*^	x	x
TLFB			x		x	x	x	x
CEQ			x		x	x	x	x
DASS-21			x		x	x	x	x
EUROHIS-QOL			x		x	x	x	x
AEs				x	x			
Cost effectiveness data			x			x	x	x
***Additional measures***								
SBQ			x					
RR I-C-R			x					
CIS			x					
Sleep measures (rMEQ + PSQI)			x^*c*^			x	x	x
Mixed-methods program evaluation	x^*d*^					^*e*^		

^a^ Primary outcome. ^b^ Randomisation occurs immediately after baseline assessment (i.e. Day 1). ^c^ rMEQ administered at baseline only. ^d^ Program evaluation continues throughout trial implementation. ^e^ Participant interviews + WAI-SR

**Table 4:** WHO Trial registration data set

Data category	Information
Primary registry and trial identifying number	ClinicalTrials.gov, NCT04713124
Date of registration in primary registry	19 January 2021
Secondary identifying numbers	E20/011/61428
Source(s) of monetary or material support	National Health & Medical Research Council (NHMRC) Clinical Trials and Cohort Studies (CTCS) Grant (186268).
Primary sponsor	National Health & Medical Research Council (NHMRC)
Secondary sponsor(s)	Eastern Health
Contact for public queries	Jasmin Grigg, MPH, PhD. jasmin.grigg@monash.edu
Contact for scientific queries	Jasmin Grigg, MPH, PhD. jasmin.grigg@monash.edu Turning Point, Richmond, Victoria, 3121 Australia
Public title	Ready2Change-Methamphetmine (R2C-M): A Randomised Controlled Trial of a Telephone-delivered Intervention to Reduce Methamphetamine Use
Scientific title	Ready2Change-Methamphetmine (R2C-M): A Randomised Controlled Trial of a Telephone-delivered Intervention to Reduce Methamphetamine Use
Countries of recruitment	Australia
Health condition(s) or problem(s) studied	Methamphetamine use disorder
Intervention(s)	Active comparator: Four to six approximately weekly sessions of R2C-M telephone-delivered intervention (50 min in duration), delivered by the same R2C-M counsellor each session. Two workbooks to facilitate counsellor-delivered exercises within sessions and one self-help booklet (as in the control group).
	Placebo comparator: Four to six telephone check-ins lasting ≤5 min and one self-help booklet.
Key inclusion and exclusion criteria	Ages eligible for study: ≥18 years Sexes eligible for study: both Accepts healthy volunteers: no
	Inclusion criteria: adult patient (≥ 18 years), mild to moderate methamphetamine use disorder, used methamphetamine on at least two occasions in the past month seeking to reduce methamphetamine use, above to provide informed consent, willing to provide details of their general practitioner or other treating physician, fluent English, literacy, regular access to a telephone and willing to provide a postal or email address.
	Exclusion criteria: currently receiving treatment for substance use disorder, requiring acute care for severe substance use disorder, requiring acute care for active suicidality or unstable psychiatric condition, a diagnosed primary psychotic disorder, pregnancy and hearing impairment profiting participation in telephone assessments.
Study type	Interventional
	Allocation: randomised intervention model. Parallel assignment masking: double-blind (participant, outcomes assessor)
	Primary purpose: treatment
	Phase n/a
Date of first enrolment	February 2021
Target sample size	196 ± 8
Recruitment status	Recruiting
Primary outcome(s)	Change in methamphetamine problem severity. Measure: Drug Use Disorders Identification Test (DUDIT). Time frame: 3 months post-randomisation
Key secondary outcomes	Change in methamphetamine problem severity. Measure: DUDIT. Time frame: 6 and 12 months post-randomisation Change in number of methamphetamine use days. Measure: Timeline Followback (TLFB). Time frame: 6 weeks and 3, 6 and 12 months post-randomisation Change in amount of methamphetamine used. Measure: TLFB. Time frame: 6 weeks and 3, 6 and 12 months post-randomisation Change in the number of DSM-5 methamphetamine use disorder criteria met. Measure: Structured Clinical Interview for DSM-5 Disorders - Research Version (SCID-5-RV). Time frame: 6 and 12 months post-randomisation Change in craving for methamphetamine. Measure: Craving Experience Questionnaire (CEQ). Time frame: 6 weeks and 3, 6 and 12 months post-randomisation Change in psychological functioning. Measure: Depression Anxiety and Stress Scale (DASS-12). Time frame: 6 weeks and 3, 6 and 12 months post-randomisation Change in psychotic-like experiences. Measure: Community Assessment of Psychic Experiences 15 (CAPE-15). Time frame: 6 weeks and 3, 6 and 12 months post-randomisation Change in quality of life. Measure: EUROHIS-QOL single item. Time frame: 6 weeks and 3, 6 and 12 months post-randomisation Change in days of other drug use. Measure: TLFB. Time frame: 6 weeks and 3, 6 and 12 months post-randomisation Difference in quality-adjusted life years (QALYs). Measure: abridged version of the 5-level EQ-5D version (EQ-5D-5L+). Time frame: over 12 months Difference in health care costs. Measure: 3Mg Health-care Resource Use Questionnaire. Time frame: over 12 months Difference in work-related losses. Measure: World Health Organization Health and Performance Questionnaire Clinical Trials Version (WHO HPQ28-Day). Time frame: over 12 months Occurrence of adverse events (AEs) and significant adverse events (SAEs). Time frame: up to 6 weeks post-randomisation

#### Follow-up assessments

Researcher 2, blind to treatment allocation, will conduct 6 week and 3, 6  and 12 month follow-up assessments by telephone, with a short message service (SMS) sent just prior to each call. Eligibility, baseline and follow-up assessment calls will take approximately 45–60 min. At least five contact attempts will be made per follow-up time point. Participants who cannot be contacted after five contact attempts will be deemed to have missing data for that time point. The research team will attempt contact again at the next follow-up, unless the participant withdraws from the study.

#### Program evaluation

Mixed-methods program evaluation will use applicable elements of the Reach, Effectiveness, Adoption, Implementation and Maintenance (RE-AIM) [62] and Consolidated Framework for Implementation Research (CFIR) [63] implementation research frameworks. A subset of participants randomised to the intervention arm will participate in semi-structured telephone-delivered interviews to understand their experiences of the program, and its implementation. Ready2Change counsellors, program and trial managers and researchers will be interviewed to seek their feedback on program implementation. Program evaluation will also be informed by trial administrative data collected during trial implementation, for example: (i) previous treatment and barriers to help-seeking of people who respond to study advertising; (ii) rate of people randomised to (a) participate in the trial from those who respond to study advertising, (b) participate in an information call and (c) participate in eligibility call; (iii) reasons for ineligibility; (iv) reasons for non-participation and lost to follow-up when available; (v) participant characteristics including health inequity factors.

### Participants

Feedback on the intervention and implementation of the Ready2Change intervention will be collected. Qualitative data from in-depth semi-structured telephone interviews will be conducted with 30 participants who received the intervention (after primary outcome data collection at 3 month post-randomisation). Participants will be purposively sampled by number of sessions completed, including those who received no Ready2Change sessions. All participants will be asked during the eligibility call if they would be willing to give feedback on the support program they received.

Staff feedback about the intervention and implementation will be obtained via in-depth semi-structured in-person or videoconferencing interviews with Ready2Change counsellors and study principal investigators. All staff members will be approached for interview and all those who consent will participate in the program evaluation.

#### Study adherence and retention

The study will use retention enhancement strategies utilised in the previous trial of the R2C program for alcohol problems [30], and as suggested in pre-trial focus groups and individual interviews with AOD service clinicians and consumers. Retention strategies include seeking verbal commitment to participate in the program, text message reminders, flexible call schedules and varying reimbursements corresponding to the importance of the data collection time-point.

#### Reimbursement

Participants will be reimbursed with supermarket vouchers as follows: AUD$20 for baseline assessment and AUD$30 per follow-up assessment. Additional reimbursement for follow-up assessments include AUD$10 for completing both 6 week and 3 month follow-ups, AUD$5 for completing 12 month follow-up or AUD$20 for completing all four follow-ups. For participation in additional study tasks, participants will be reimbursed as follows: AUD$20 for completing the CIS cognitive assessment task and AUD$10 for participating in a program feedback interview.

#### Concomitant and post-trial care

The risks of harm to participants in this study are anticipated to be minor and no compensation for harm is deemed necessary. Although receiving treatment for substance use problems is an exclusion criterion for this study, participants are not restricted from seeking other treatment for MA or other substance use problems after they begin the trial. Throughout the study, participants needing further support will be referred to additional services. The research team will monitor the number of participants who receive or are referred to further AOD services or escalated to the study PI for clinical review.

### Data collection

An electronic case report form (eCRF) will be completed for each participant using the secure, web-based application REDCap, which will contain all eligibility and study data (listed in Table 2). REDCap is hosted on a secure server and managed by Eastern Health Information Technology Services with individual access via a secure login. Only approved members of the research team will have access to the eCRFs. As part of verbal consent, and outlined in the Participant Information Sheet, participants will be asked to provide their consent for their re-identifiable data (i.e. their name will not be attached to the data, but only by the unique participant code assigned to both personal information and data, making the data technically re-identifiable) to be used in any future, related research projects conducted by the research team or for student projects. Separate ethics approval will be sought for any subsequent, related project requesting to use these data. Security of participant data will be upheld at all times, and persons working with the research team on subsequent, related studies will not ever have access to participants’ identifiable data.

### Data retention

All data collected during this study will be retained by the investigator for a period of at least 5 years as outlined in the Australian Code for the Responsible Conduct of Research [64].

### Study oversight

#### Trial monitoring

The trial’s Chief Investigators will perform the function of a Trial Management Committee (TMG), as they have the expertise necessary to oversee all aspects of the conduct of the trial (i.e. capacity to monitor compliance with the protocol, monitor compliance with ethical and clinical governance, provide standardised training and other means of quality control, oversee trial arm fidelity, monitor adverse events and provide leadership to the research team). Data audits will be conducted at periodic intervals (i.e. every 6 months), led by the trial manager. Regular liaison between the principal investigator, study clinician and research team will occur to permit discussion of day-to-day trial progress, participant eligibility and any potential concerns. Any protocol amendments decided by the investigators (e.g. changes to eligibility criteria, outcomes, analyses) will be communicated to the ethics committees and clinical trials register.

#### Treatment adherence and integrity

R2C-M counsellors will undergo training focusing on competence and adherence to the R2C-M intervention and research procedures. All sessions will be recorded and an independent researcher will rate the fidelity (i.e. adherence and competence) of an intervention session for 20% of the active sample using random start systematic sampling. Adherence to intervention elements will also be monitored by R2C-M counsellors via a standard checklist. Researcher 1 (who conducts control condition check-in calls) will be trained to provide information on further AOD support and use a script to ask about participants’ use of the information booklet (i.e. to ensure that participants in the control condition do not inadvertently receive individualised support). Supervision of Researcher 1 will occur to prevent “drift” (e.g. call duration records will be checked intermittently).

#### Adverse events

It is recognised that adverse effects can arise from the delivery of psychological interventions in clinical trials [65]. In this trial, adverse events (AEs) and serious adverse events (SAEs) that could be related to methamphetamine use or psychological intervention will be systematically collected during the intervention period and at 6 weeks post-randomisation, for both R2C-M and control conditions. Participants will be asked if they experienced any negative effects following their previous telephone support call and whether they have been hospitalised since their last call. Examples of “negative effects” will be provided if requested and prompts include significantly increased distress, significantly increased MA cravings or the issue for which the participant was looking for help got a lot worse. Unexpected harms will be collected as notes on the participants REDCap file if volunteered by the participant. Participants will also be encouraged to contact the research team if they are concerned about an adverse event. Any AEs or SAEs occurring during the course of this study, whether or not they are deemed to be related to participation in this study, will be followed rigorously, and in conjunction with the participant’s general practitioner as appropriate (general practitioner or practice contact details will be collected at baseline, if the participant has a current general practitioner or practice). All adverse events will be reported to the approving ethics committee and included in subsequent peer-review publications.

#### Participant assessed as a risk of suicide

If a participant is assessed as being at risk of suicide, referral to appropriate support is immediate. Researchers will undertake suicide intervention skills training, to ensure they are equipped with the skills to respond to suicide risk, particularly when contingencies may be required (e.g. assessing urgency—evaluating the need to keep the participant on the phone; managing the call when risk is immediate). R2C-M counsellors are well-trained and experienced in the management of suicide risk and will follow established clinical risk assessment and management guidelines.

#### Participant withdrawal and discontinuation

Participants’ right to withdraw from the trial without consequence will be outlined during the consenting process and in the Participant Information Sheet. Participants can withdraw their consent verbally or in written form (i.e. email or text message correspondence), with the option to remove all previously collected data or just remove consent for further data collection. No further contact with the participant will be initiated by the research team upon their withdrawal from the study. In instances where it has been identified that a participant meets exclusion criteria during the study (e.g. active suicidality), and/or that it is not in the best interests of the participant to remain in the study, the Principal Investigator or Study Clinician will decide whether to withdraw the participant from the trial. The reasoning for this will be explained to the participant and they will be offered information on accessing other support. No further data collection will occur, with the exception of the details regarding adverse events.

### Statistical methods

#### Sample size estimates

We aim to randomise between 188 and 204 participants to this study (i.e. total *N* = 196 ± 8, subjects per study arm = 98 ± 4). This was calculated using the Genstat [66] power procedure. The primary outcome measure (DUDIT score 3 months post-baseline) can range from 0 to 40 and will be analysed via a linear mixed model. Using data from our pilot work [20], we found that the between-subject variance component in DUDIT score was 21, the within-subject variance component was 63 and the estimated improvement (decline) in DUDIT score was 17 (SE = 1.6). We estimate that by 3 months there will be an improvement of at least 16 in the R2C-M arm and that the control arm could exhibit an improvement of up to 10. With 75 evaluable subjects in each treatment arm, the study will have 90% power to detect this difference in improvement using an *F*-test conducted at the 5% significance level. If these conjectured improvements by 3 months are not durable and, for example, deteriorate by 50% at 6 months, and return, on average, to baseline values by 12 months, then this treatment-by-time interaction scenario will be detected with at least 90% power. The initial target sample size of 188 comprises 75 per arm, inflated to 94 per arm to allow for approximately 20% drop-out, which is based on the attrition rates reported in other treatment [67] and helpline [68] research with AOD cohorts using 12 month endpoints. Provision has been made to increase the recruitment target, based on the 3 and 6 month attrition rates observed after recruitment and data collection commenced (after 12 months of data collection with this complex cohort, 3 and 6 month attrition rates were higher than anticipated). As such, the target sample size was increased from 188 subjects, to up to 204 participants (i.e. an additional 8 subjects based on the 3 month attrition rate; or an additional 16 subjects based on the 6 month attrition rate). Based on our experience with social media advertising for alcohol and other drug treatment trials, it is estimated that 10–12 participants will be recruited per month over 18 months.

#### Statistical analysis plan

Data will be collated, cleaned and validated using programed edit checks, in a database that will be locked prior to the unblinding of the statistician for the primary analysis. The primary analysis will take place after all subjects, not known to have withdrawn or not deemed lost to follow-up, have had their 12 month assessments and will be based on the intention-to-treat principle (i.e. subjects’ data are analysed as randomised and as stratified). A “per-protocol” sensitivity analysis will be restricted to those subjects with at least one follow-up assessment and, for subjects randomised to the R2C-M arm, participation in at least two telephone counselling sessions. Previous research delivering the R2C program to people with alcohol use disorder found exposure of ≥2 sessions yielded a reduction in alcohol use severity compared to a control arm [31]. The first R2C session focuses on a clinical assessment and identifying treatment goals and the second session is when a therapeutic dose is received. As such, exposure to ≥2 sessions is considered “as-treated” for the per-protocol analysis. Additional sensitivity analyses will include a covariate for the number of structured telephone counselling sessions [1 to 6] in which subjects, in the R2C arm, participated. The repeated measurements of the outcome variables will be analysed by fitting linear mixed models using restricted maximum likelihood (REML)—this will allow the most suitable variance-covariance model for the repeated measures to be selected, using Akaike’s Information Criterion, and commonality of nonlinear trends over time to be explored via splines. The *F*-test will be used to test for an overall group by time interaction and the primary comparison, between groups, of their changes from baseline to 3 month follow-up will be based on a *t*-test of the corresponding interaction contrast—this *t*-test will utilise the predicted means and their variance-covariance matrix which are recovered from the fitted mixed model. Diagnostic plots of residuals will be assessed and, if deemed necessary, variance-stabilising transformations such as the empirical logistic transformation will be applied to the outcome variables, and inferences will be based on the analyses conducted on the transformed scale. In a series of exploratory analyses, mixed models with covariates for gender, illicit drug use, extent of exposure to the intervention, differences due to assigned counsellor, exposure to other treatments/programs and baseline levels of MA use, psychological distress, depression, anxiety and stress will be fitted, including their interactions with treatment group, in order to identify moderating factors. The complete list of candidate covariates and details of the analyses will be specified in a Statistical Analysis Plan that will be reviewed and approved by a Study Management Committee prior to database lock. No interim analyses will be conducted and there are no plans to halt data collection before completion. Analyses will be conducted using the most appropriate procedures in GenStat, R and Stata.

#### Cost-effectiveness analysis

The economic evaluation will assess the mean incremental costs and mean incremental benefits of treatment of R2C-M compared to control. Benefits will be measured as quality-adjusted life years (QALYs). Incremental QALYs will be measured by the between-group difference in mean EQ-5D-5L+ score over 12 months. A health system perspective on costs will be taken and will include resource use incurred in the delivery of telephone intervention as well as health services irrespective of payment source. Health care costs will be calculated from the utilisation data and average unit costs for each item. Running costs will be included, but not the costs of training in the primary analysis. In a supplementary analysis, we will model the potential cost-effectiveness using a broader societal perspective and include estimates of the cost of work-related losses using the WHO HPQ28-day, crime and interpersonal related harms associated with MA use from literature sources.

Cost-effectiveness analysis results will be presented as the mean net benefits of treatment across a range of hypothetical money values of QALYs, with 95% CIs and a one-sided *p*-value calculated using non-parametric bootstrapping. Net benefit estimates will be based on the between-group difference in the means cost and outcome over the 12 months estimated using separate regression analyses controlling for baseline values and the stratification variable. A generalised linear regression model, with an appropriate choice of distribution, will be used to account for any skewness in the cost data. Multiple imputation will be used to address the uncertainty of the estimates due to missing observations. A secondary analysis will estimate a per-protocol cost-effectiveness of the intervention adjusted for non-adherence. Instrumental variable estimation will be used with the randomisation group as the instrument for adherence, defined as at least two telephone counselling sessions [69].

#### Dissemination and translation plan

Dissemination of findings to the research community will be via peer-reviewed publications and conference presentations. Chief Investigators will meet near the end of the trial to finalise and implement the research dissemination plan and authorship. There are no plans to engage paid professional writers outside the study team. Participants will be informed they can access the Turning Point website for a summary report of the results at the trial’s end.

## Discussion

The substantial impacts of MA use across Australia and the low rates at which individuals with MA use disorder seek treatment highlight the need for accessible, evidence-based interventions that can reduce MA use and related harms, and decrease the burden on communities and health services [3, 6]. Telephone-delivered interventions overcome many of the individual and structural barriers to seeking treatment faced by individuals with MA use problems [8]. This is the first RCT internationally to examine the effectiveness of a telephone-delivered intervention for mild-to-moderate MA use disorder; this model is anticipated to reach a group who are unlikely to access mainstream services, to offer intervention earlier in the continuum of problematic use and prevent the development of chronic MA use and related problems. Study outcomes are also likely to inform the delivery of alternative interventions for a range of other conditions, particularly those where help-seeking is low, stigma is high and/or early intervention is a priority (e.g. other illicit drug use, gambling, mental health disorders).

Feasibility of this trial and its execution is high; previously demonstrated with people seeking to reduce their alcohol use, the proposed intervention has also already been piloted within an existing service, and the study will harness the success of an ongoing partnership between universities and a lead agency for the provision of alcohol and drug treatment across Australia. With a research-to-practice gap evident in all health service delivery, wherein there is a significant lag time to the implementation of treatments shown to be effective in research, the existing link between the research team and a national treatment service is a major strength of this study, allowing the findings to be quickly disseminated. As such, the outcomes of this project are expected to make a significant contribution to the health and well-being of a population who face substantial barriers accessing treatment services, as well as reducing the burden on and generating substantial cost savings for the health system and broader community.

People with MA use disorder form a large, highly stigmatised group with significant mortality and morbidity, for whom new and effective treatments are urgently needed. If found to be effective, the R2C-M model will fill a significant public need by providing an innovative, cost-effective, accessible means of early intervention for reducing MA use and associated problems.

## Trial status

This trial is at protocol version 5, dated 1 September 2022. Recruitment of participants commenced on 4 February 2021 and is expected to be completed by February 2023 (with the last 12 month follow-up to be completed by February 2024).

### Trial registration data set

See Table 4.

## Supplementary Information

**Additional file 1.** SPIRIT Checklist for Trials.

**Additional file 2.** Participant Information Sheet and Consent Form; Telephone intervention study - Adult providing own consent.

## Data Availability

Only approved members of the research team will have access to the final trial data set. There are no plans to grant public access to the participant-level dataset as we do not have ethics approval or participant consent to do so. There are no plans to grant public access to the statistical code, but we will provide public access to the full research protocol and statistical analyses plan via ClinicalTrials.gov NCT04713124.
