# Supplementary material for: A structured, telephone-delivered intervention to reduce methamphetamine use: study protocol for a parallel-group randomised controlled trial
Source: Trials. 2023 Mar 29;24:235. doi: 10.1186/s13063-023-07172-9 (PMC10052286; doi:10.1186/s13063-023-07172-9)
Supplement: Supplementary file 2 — Additional file 2. Participant Information Sheet and Consent Form; Telephone intervention study - Adult providing own consent. [file 13063_2023_7172_MOESM2_ESM.docx]

**Participant Information Sheet**

**Telephone Intervention Study** - *Adult providing own consent*

| **Title**  **Short Title** | A randomised controlled trial of a telephone-delivered intervention to reduce methamphetamine use  Ready2Change-Methamphetamine (R2C-M) |
| --- | --- |
| **Project Sponsor** | Eastern Health |
| **Principal Investigator** | Professor Dan Lubman |
| **Location** | Turning Point, VIC |

**Part 1 What does my participation involve?**

**1 Introduction**

You are invited to take part in a study comparing two types of telephone support programs to assist in reducing methamphetamine use. The telephone support runs for six sessions, is delivered by the same support caller at times convenient to you, and can be accessed from anywhere in Australia. You have been invited to take part because you would like to reduce how much or how often you take methamphetamine, and you have responded to study advertising or have otherwise heard about this study from a helpline counsellor or your healthcare provider.

This Participant Information Sheet tells you about the research study. It explains what is involved in taking part. Knowing what is involved will help you decide if you want to take part in the research.

Please read this information carefully. Ask questions about anything that you don’t understand or want to know more about. Before deciding whether or not to take part, you might want to talk about it with a relative, friend or local health worker.

**Participation in this research is voluntary. If you don’t wish to take part, you don’t have to. Whether or not you choose to take part will not affect any usual care you receive.**

If you would like to participate, you will be asked to provide your consent verbally. By providing verbal consent to participate, you are telling us that you:

• Understand what has been explained to you

• Consent to take part in the research project

• Consent to have the assessments that are described

• Consent to the use of your personal and health information as described

Please save a copy of this Participant Information Sheet for any future reference.

This study is being conducted by Turning Point (part of Eastern Health). Turning Point is a national addiction treatment centre, dedicated to providing high quality, evidence-based treatment to people adversely affected by alcohol, drugs and gambling, integrated with world-leading research and education. The study also involves clinicians and researchers from Monash University and Deakin University, who will be working in collaboration with Turning Point.

**2 What is the purpose of this research?**

The purpose of this research is to compare two types of telephone support programs to assist in reducing methamphetamine use. If you choose to take part you will be assigned by chance to receive one of the two telephone support programs, wherein you will receive six telephone calls by the same support caller, as well as supporting materials by email/post.

This study aims to recruit 188 participants from across Australia: 94 participants will be randomly allocated to each of the two telephone support programs.

**3 What does participation in this research involve?**

This entire study is conducted by telephone. Figure 1. shows the steps of participation in this study. As well as receiving six telephone support calls, a researcher will check in with you by telephone over a 12-month period to ask you some questions, for example, about your quality of life and substance use.

*Figure 1***.** Steps of Participation

**Eligibility call**

Before you can start the study, a member of the study team will talk to you by telephone about the study. If you are interested in participating, they will ask you for your contact details and some basic demographic information (e.g. your gender, date of birth), and will ask you some questions in order to determine if you meet certain requirements for participating (e.g. current substance use, mental health). This telephone call will take approximately 1 hour to complete.

You are eligible to participate if you:

- Are 18 years or older
- Are identified as having a mild-to-moderate methamphetamine use disorder
- Have used methamphetamine at least twice in the past month
- Are seeking to reduce your methamphetamine use
- Are able to provide informed consent, and comply with the requirements of the study (i.e. be willing to take part in telephone support sessions, as well as researcher check-ins, over a 12-month period)
- Are willing to provide the contact details of your regular GP or other health practitioner (if you have one)
- Speak English as your first language or fluent
- Are educated to high school level
- Have regular access to a telephone
- Have a postal/email address by which to receive support materials

You are **not** able to participate in this research if you:

- Are currently receiving treatment for a substance use disorder (e.g. medically supervised detoxification, residential rehabilitation, drug counselling, medication). This only applies at study entry and does not prevent you from seeking additional support during your participation in the study.
- Require acute care for a severe substance use disorder, active suicidality or an unstable psychiatric condition (as assessed during the screening call)
- Have been diagnosed with a primary psychotic disorder (schizophrenia, schizoaffective disorder, or bipolar disorder)
- Are currently pregnant
- Have a hearing impairment that would prohibit participation in this telephone study

If you are eligible to participate in this study, and decide that you want to take part, you will be asked to provide your verbal consent to participate before any of the study-related activities commence. Before you provide verbal consent to participate, the study team member will go through the information outlined in this Participant Information Sheet (e.g. procedures, risks, benefits), and you will be able to discuss the study and ask questions.

**Baseline and program allocation call**

The baseline call will typically occur within one week of the eligibility call, and will take approximately 1 hour to complete. During this call, you will be asked some questions about your mental health, quality of life, substance use, use of health services, and work performance.

During this call, you will be assigned by chance to receive one of the two different telephone support programs being evaluated. You have a 1 in 2 chance of being in each group. Neither you nor the researcher conducting the later follow-up assessments will know which group you are in. The results will be compared to see if one telephone program is better.

Before the baseline call ends, you will be given an overview of the telephone support you will receive, and the first session will be scheduled for you (typically within the next week), on a day and at a time that suits you. Some support materials will be posted/emailed to you. Hard copy information will be posted to you in an unmarked envelope. Please remember to store hard copies of materials sent by us in a secure space at home if you want to maintain discretion about your participation in this study.

**Telephone Support Calls**

You will receive six telephone calls by the same support caller, approximately weekly on pre-arranged days and at times that suit you best. If we cannot reach you as arranged, we will attempt to reach you at a later time. We will send SMS reminder messages of pre-arranged telephone support calls.

**Follow-up Calls**

After you have completed the telephone support program, a researcher will check in with you four times over the rest of the 12-month study period to see how you are going, and ask you some questions about your mental health, quality of life, substance use, use of health services, and work performance. Follow-up assessments will occur:

- 6 weeks after program allocation (45 minutes)
- 3 months after program allocation (1 hour)
- 6 months after program allocation (1 hour)
- 12 months after program allocation (1 hour)
- You may also be asked to take part some extra activities, i.e. computer cognitive task, or to provide program feedback.

**4 Reimbursement**

There are no costs to you for participating in this research project. You will be reimbursed with vouchers (e.g. supermarket voucher, emailed or posted) for your time taken to complete each research call, as follows:

- $20 reimbursement for completing the baseline call
- $30 reimbursement for completing the 6-week follow-up call
- $30 reimbursement for completing the 3-month follow-up call
- $30 reimbursement for completing the 6-month follow-up call
- $30 reimbursement for completing the 12-month follow-up call
- If you consent to, and are selected for the additional components of the study, you will be reimbursed for your time (computer cognitive task - $20; program feedback - $10).

**In addition to these standard reimbursements, as an additional incentive:**

- Participants who complete the first two follow-up calls (i.e. 6-week and 3-month calls) will receive a $10 bonus supermarket voucher
- Participants who complete all four follow-up calls (i.e. 6-week, 3-month, 6-month, and 12-month calls) will receive an additional $20 bonus voucher
- Participants who have not completed all four follow-up calls but who complete the final 12-month follow-up call will receive a smaller $5 bonus voucher

**The maximum additional incentive a participant can obtain is $30**

**5 Do I have to take part in this research project?**

Participation in any research project is entirely voluntary. If you do not wish to take part, you do not have to. If you decide to take part and later change your mind, you are free to withdraw from the project at any stage. Your decision whether to take part or not, or to take part and then withdraw, will not affect any routine care or your relationship with Turning Point or collaborating organisations.

**6 What are the alternatives to participation?**

You do not have to take part in this research project to receive treatment at Turning Point, including their telephone (e.g. DirectLine) and online support services (e.g. Counselling Online). Other treatment options and telephone and online services are available to help with substance use problems, and you can see your GP to discuss other alternative treatment options.

**7 What are the possible benefits of taking part?**

We cannot guarantee or promise that you will receive any benefits from this research. However, multiple session support programs have been shown to be effective in reducing methamphetamine use problems. Multiple session support programs methamphetamine use problems that are delivered by telephone are less well researched, however they hold potential for reducing problems related to methamphetamine use.

**8 What are the possible risks and disadvantages of taking part?**

The risks of harm and discomfort to participants are anticipated to be relatively minor. Some participants may find that discussing their methamphetamine use triggers cravings for methamphetamine or psychological distress. However, our experience from previous studies suggests that this is uncommon and any distress is usually minor and transient. While it is unlikely that you will experience any adverse effects from participating in one of the telephone support programs being evaluated, the research team will be looking out for possible adverse effects throughout your participation in the program you are allocated to.

**9 What if new information arises during this research project?**

Sometimes, during the course of a research project, new information becomes available about the treatment/intervention methods being studied. If this happens, the research team will tell you about it and discuss with you whether you want to continue in the research project. If you decide to withdraw, the research team will provide you with information on alternative treatment approaches you might like to consider discussing with your GP. If you decide to continue to participate in this study, you will be asked to sign an updated consent form.

It may be that, on receiving new information, the research team might consider it to be in your best interests to withdraw you from the research project. If this happens, they will explain the reasons and provide you with information on alternative treatment methods and encourage you to see your GP to discuss use of these alternatives.

**10 Can I have other treatments during this research?**

Whilst you cannot be receiving other treatment for methamphetamine use problems when you enter into this research project, you are not restricted from seeking other treatment for methamphetamine use problems (e.g. outpatient/inpatient treatment, drug counselling, medication, online support through Counselling Online) after you begin this study. You will be asked about your engagement in any additional treatment throughout your participation in this study, and it is important to tell the research team about any other treatments you may be receiving, or medications you may be taking, for methamphetamine use problems (including over-the-counter medications). You should also tell the research team about any changes to these during your participation in the research project.

**12 What if I withdraw from this research project?**

If you do consent to participate, you may withdraw at any time. If you decide to withdraw from the study, please notify a member of the research team before you withdraw. You can withdraw from this study by phoning or texting the researcher or support caller you are in contact with.

If you decide to withdraw from the project, the research team will not collect additional personal information from you, although personal information already collected will be retained to ensure that the results of the research project can be measured properly and to comply with law. You should be aware that data collected up to the time you withdraw will form part of the research project results. If you do not want your data to be included, you must tell the researchers when you withdraw from the research project.

**13 What happens when the research project ends?**

A summary of grouped findings (i.e. in a way that you will not be identifiable) will be available on the Turning Point website (turningpoint.org.au).

The telephone support program you receive during this study will not continue to be provided at the end of your participation. You will be able to keep the support materials provided to you. Other services provided by Turning Point, including their telephone (e.g. DirectLine) and online support services (e.g. Counselling Online) will be available as usual, and other treatment options are available to help with methamphetamine use problems. You can see your GP to discuss further treatment options as required.

**Part 2 How is the research project being conducted?**

**14 What will happen to information about me?**

By providing verbal consent, you consent to the research team collecting and using personal/identifiable information about you for the research project. Any information that we collect which can identify you will remain confidential. The information collected in this study will remain re-identifiable. This means information which can identify you (e.g. your name, date of birth) will be separated from the study data you provide, linkable only by an obscure code.

Your personal/identifying information will be kept separately from your study data and only the members of the research team conducting the intervention phase and follow-up assessments will have access to this information, and the codes linking the two. All of your information will be kept strictly confidential and will only be disclosed with your permission, except when it is subject to inspection (for the purposes of verifying the procedures and the data) by the authorised representatives of the Human Research Ethics Committee (HREC) of Eastern Health, or as required by law (e.g., in response to a court order). You will be notified if such disclosure is ever required.

Telephone calls wherein we collect your baseline and follow-up information will not be recorded. The telephone calls you receive as part of the methamphetamine program may be recorded, but only with your permission. You will have the opportunity to opt out of having any call recorded, before the call proceeds.

The collection, use and disclosure of your health and personal information is governed by the Health Records Act 2001 (Vic) (HR Act) and the Privacy and Data Protection Act 2014 (VIC) (PDA Act), respectively. The individual data that we collect, by law, has to be stored for at least 5 years after the end of the project. The data will be locked in secure storage at Turning Point until it is destroyed which will be at a time soon after 31^st^ December 2027. You have the right to access the information collected and stored by the researchers about you. Please contact the research team if you would like to access your information.

It is anticipated that the results of this study will be published and/or presented in a variety of forums (e.g. journal articles, conferences). In any publication and/or presentation, information will be provided in aggregate format; that is, in such a way that you cannot be identified, to protect your privacy and confidentiality.

**Consent to future use of data**

When providing verbal consent, you will be asked to provide consent for your re-identifiable data to be used in any future, related research projects conducted by the research team or for student projects. Your identifiable information will not ever be used in subsequent, related projects. Separate ethics approval will be sought for any subsequent, related project requesting to use these data. Any future use of your data will be in aggregate form (i.e. in such a way that you cannot be identified). Persons working with the research team on subsequent, related studies will not ever have access to your personal information, identifiable data, nor the spreadsheet containing re-identifiable information.

**15 Complaints and compensation**

If you have any complaints about this research you should direct these to the project team whose contacts details can be found below.

If you suffer any distress or psychological injury as a result of this project, you should contact the project team as soon as possible. You will be assisted with arranging appropriate treatment and support.

We do not anticipate any need for medical treatment as a result of this project. However, if you require any medical treatment as a result of this research project, you should contact the study team as soon as possible and you will be assisted with arranging appropriate assistance. If you are eligible for Medicare, you can receive any medical treatment required to treat the medical complication, free of charge, as a public patient in any Australian public hospital.

**16 Who is organising and funding the research?**

This research project is being conducted by a team of clinicians and researchers led by Professor Dan Lubman (Turning Point, part of Eastern Health; Monash University). It is being funded by a Project Grant from the National Health & Medical Research Council (NHMRC).

No member of the research team will receive a personal financial benefit from your involvement in this research project (other than their ordinary wages).

**17 Who has reviewed the research project?**

All research in Australia involving humans is reviewed by an independent group of people called a Human Research Ethics Committee (HREC).

The ethical aspects of this research project have been approved by the HREC of Eastern Health. This project will be carried out according to the National Statement on Ethical Conduct in Human Research (2007). This statement has been developed to protect the interests of people who agree to participate in human research studies.

**18 Further information and who to contact**

The person you may need to contact will depend on the nature of your query. If you want any further information concerning this project or if you have any problems which may be related to your involvement in the project, you may phone the Trial Manager, Dr Jasmin Grigg on (03) 8413 8723 or contact her by email: [jasmin.grigg@monash.edu](mailto:jasmin.grigg@monash.edu).

If you have any complaints about any aspect of the study or the way in which it is being conducted, you may contact the Eastern Health Office of Research and Ethics on (03) 9895 3398 or via email: [ethics@easternhealth.org.au](mailto:ethics@easternhealth.org.au)

**Seeking Verbal Consent** - *Adult providing own consent*

| **Title** | A randomised controlled trial of a telephone-delivered intervention to reduce methamphetamine use (Ready2Change-Methamphetamine; R2C-M) |
| --- | --- |
| **Project Sponsor** | Eastern Health |
| **Principal Investigator** | Professor Dan Lubman |
| **Location** | Turning Point, VIC |

**Declaration by Participant**

I have had the participant information explained to me, and have been provided with a copy of this Participant Information Sheet (either by web link, email or post). **Y / N**

I understand the purposes, procedures and risks associated with this project. **Y / N**

I have had an opportunity to ask questions and I am satisfied with the answers I have received. **Y / N**

I freely agree to participate in this research project as described and understand that I am free to withdraw at any time during the project without affecting my future care. **Y / N**

I freely agree to allow the research team to contact me by telephone as described for this research project.
**Y / N**

I would be willing to provide feedback about the support program I received, if I am selected to do so. **Y / N**

I am willing to participate in the additional computer cognitive task. **Y / N**

I consent to my information, collected in this research, being used in future, related research projects. I understand that subsequent, related projects will not ever use my identifiable information, but may use my data in aggregate form (i.e. in such a way that you cannot be identified). **Y / N**

Do you provide your verbal consent to participate in this research project? **Y / N**
